# A moderate elevation of circulating levels of IGF-I does not alter ErbB2 induced mammary tumorigenesis

**DOI:** 10.1186/1471-2407-11-377

**Published:** 2011-08-25

**Authors:** Robert K Dearth, Isere Kuiatse, Yu-Fen Wang, Lan Liao, Susan G Hilsenbeck, Powel H Brown, Jianming Xu, Adrian V Lee

**Affiliations:** 1 Lester and Sue Smith Breast Center, Baylor College of Medicine, One Baylor Plaza, Houston, TX, 77030, USA; 2 Department of Biology, University of Texas-Pan American, 1201 West University Drive, Edinburg, TX, 78539, USA; 3 Department of Molecular and Cellular Biology, Baylor College of Medicine, One Baylor Plaza, Houston, TX, 77030, USA

## Abstract

**Background:**

Epidemiological evidence suggests that moderately elevated levels of circulating insulin-like growth factor-I (IGF-I) are associated with increased risk of breast cancer in women. How circulating IGF-I may promote breast cancer incidence is unknown, however, increased IGF-I signaling is linked to trastuzumab resistance in ErbB2 positive breast cancer. Few models have directly examined the effect of moderately high levels of circulating IGF-I on breast cancer initiation and progression. The purpose of this study was to assess the ability of circulating IGF-I to independently initiate mammary tumorigenesis and/or accelerate the progression of ErbB2 mediated mammary tumor growth.

**Methods:**

We crossed heterozygous TTR-IGF-I mice with heterozygous MMTV-ErbB2 mice to generate 4 different genotypes: TTR-IGF-I/MMTV-ErbB2 (bigenic), TTR-IGF-I only, MMTV-ErbB2 only, and wild type (wt). Virgin females were palpated twice a week and harvested when tumors reached 1000 mm^3^. For study of normal development, blood and tissue were harvested at 4, 6 and 9 weeks of age in TTR-IGF-I and wt mice.

**Results:**

TTR-IGF-I and TTR-IGF-I/ErbB2 bigenic mice showed a moderate 35% increase in circulating total IGF-I compared to ErbB2 and wt control mice. Elevation of circulating IGF-I had no effect upon pubertal mammary gland development. The transgenic increase in IGF-I alone wasn't sufficient to initiate mammary tumorigenesis. Elevated circulating IGF-I had no effect upon ErbB2-induced mammary tumorigenesis or metastasis, with median time to tumor formation being 30 wks and 33 wks in TTR-IGF-I/ErbB2 bigenic and ErbB2 mice respectively (p = 0.65). Levels of IGF-I in lysates from ErbB2/TTR-IGF-I tumors compared to ErbB2 was elevated in a similar manner to the circulating IGF-I, however, there was no effect on the rate of tumor growth (p = 0.23). There were no morphological differences in tumor type (solid adenocarcinomas) between bigenic and ErbB2 mammary glands.

**Conclusion:**

Using the first transgenic animal model to elevate circulating levels of IGF-I to those comparable to women at increased risk of breast cancer, we showed that moderately high levels of systemic IGF-I have no effect on pubertal mammary gland development, initiating mammary tumorigenesis or promoting ErbB2 driven mammary carcinogenesis. Our work suggests that ErbB2-induced mammary tumorigenesis is independent of the normal variation in circulating levels of IGF-I.

## Background

IGF-I has the characteristics of both a circulating hormone and a tissue growth factor. While numerous studies have focused on the autocrine and/or paracrine ability of IGF-I to regulate mammary gland development and tumorigenesis [1], only a few have focused on the role of circulating IGF-I [2]. Although it is known that circulating levels of IGF-I vary considerably within the normal population, meta-analysis of several studies have shown that elevated serum IGF-I levels are associated with increased risk of breast cancer in premenopausal women [3]. Mammographic density is strongly related to breast cancer risk [4] and evidence supports a positive correlation between circulating IGF-I levels and mammographic density [5]. Supporting this, the IGF-I axis correlates with birth weight, height, and parity, all which have been show to be contributing breast cancer risk factors [6,7]. Increased IGF-I signaling has also been linked to trastuzumab resistance in ErbB2 positive breast cancer [8] and blocking both the IGF-I receptor (IGF-IR) and the ErbB2 receptor (ErbB2-R) inhibits ErbB2 driven breast cancer cell growth [9]. Conversely, antiestrogens that are effective in the treatment and prevention of breast cancer have been consistently found to lower serum IGF-I levels [10].

The idea that circulating IGF-I may regulate breast cancer is persuasive, especially taking into account that IGF-I, mediated by the endocrine actions of growth hormone (GH), plays a vital role in regulating the developing mammary gland [11]. The mammary gland can proliferate in response to IGF-I both in organ culture [12], and by treatment of mice with an implant containing IGF-I in the mammary gland [13]. Conversely, it has been shown that there is very limited growth of IGF-IR-null mammary epithelium [14]. In addition, IGF-I-null mice have severely retarded mammary ductal development and branching [11]. These and other studies support a model whereby GH acts upon mammary stroma which produces IGF-I to stimulate pubertal mammary ductal outgrowth in a paracrine manner. However, recent studies in mice with only circulating IGF-I and no local production have shown that endocrine IGF-I can also support mammary ductal growth [15,16]. We have previously shown that circulating IGF-I, via tail vein administration, results in activation of IGF signaling in the mammary gland [17]. Conversely, using a novel GH antagonist, pegvisomant, we showed that blocking GH action, which results in a lowering of serum IGF-I levels can block IGF-I signaling in the mammary gland and results in a delay of mammary gland development [18].

It has been shown that *little *(lit/lit) mice, which have only 10% of circulating IGF-1 levels, displayed a significant reduction of growth of human MCF-7 cell xenografts [19]. Similarly, deletion of the IGF-I gene in the liver, resulting in an 80% reduction in circulating IGF-I, delays chemically and transgenically-induced mammary tumorigenesis and metastasis[20]. As compelling as these studies may be in supporting the notion that reduced circulating IGF-I may limit breast cancer initiation and progression, the 10% level of circulating IGF-I doesn't mimic epidemiologic studies.

There are few mouse models that show moderate changes in circulating IGF-I that are comparable to human variation. We originally developed TTR-IGF-I transgenic mice which overexpress IGF-I mRNA exclusively in the liver and show a moderate but significant increase in circulating levels of IGF-I [21]. Importantly the 35% increase in circulating IGF-I demonstrated in this model is comparable to the 31% increase in circulating IGF-I levels shown to increase breast cancer risk in premenopausal women [22]. The increase in our model is significantly less than the 2.5-fold increase seen in a similar TTR-IGF-I model recently reported by Wu *et al. *[15]. To determine if increased levels of IGF-I can initiate mammary tumorigenesis and/or promote ErbB2-induced tumorigenesis, we crossed TTR-IGF-I transgenic mice with the well characterized MMTV-ErbB2 transgenic mice. Increased circulating IGF-I did not alter pubertal mammary gland development or total body composition. We show that although TTR-IGF-I and bigenic mice had a 35% increase in circulating total IGF-I compared to ErbB2 transgenic and control mice, IGF-I alone was insufficient to cause mammary tumorigenesis. Furthermore, the elevation of systemic IGF-I had no effect on ErbB2-induced mammary tumorigenesis. Analysis of ErbB2-intiated tumors revealed no major effect of increased circulating IGF-I on tumor type (solid adenocarcinomas) or mammary gland signaling. This is the first study using a transgenic animal model that mimics the variation of normal circulating levels of IGF-I in epidemiological studies. Our studies show that elevated circulating IGF-I has no effect on normal mammary gland development or ErbB2-induced mammary tumorigenesis.

## Methods

### Animals and Experimental Design

All animals were housed under controlled conditions of temperature (23°C), lights (lights on: 0600 h; lights off: 1800 h) and ad libitum access to food (Harland Teklad Diet, Madison, WI) and tap water. The care and handling of animals used in this study followed the guidelines established by the National Institutes of Health (NIH) and all humane procedures were pre-approved by the University Laboratory Animal Care Committee (ULACC).

Homozygous TTR-IGF-I (tg/tg) transgenic females on a C57/Bl6 background were developed and characterized previously [21]. Due to the known ability of the C57/Bl6 genetic background to inhibit oncogene-induced mammary tumorigenesis[23], homozygous TTR-IGF-I mice were first backcrossed to FVB/N mice for six generations to create homozygous TTR-IGF-I/FVB/N animals in order to cross these animals with the ErbB2 mouse model MMTV-c-Neu FVB/N. MMTV-c-Neu FVB/N mice (Jackson Laboratories) are based on a mammary specific overexpression of the ErbB2 receptor (a frequently amplified oncogene in human breast cancer), which results in mammary specific tumorigenesis[24].

Homozygous TTR-IGF-I and homozygous MMTV-c-Neu FVB/N (further referred to as MMTV-ErbB2) mice were crossed to FVB/N wild type mice to produce heterozygous transgenics. For mammary gland developmental studies heterozygous TTR-IGF-I (tg/wt) FVB females and age-matched wild type FVB/N littermates (controls) were sacrificed at 4 weeks, 6 weeks and 9 weeks of age. Blood was collected and mammary glands processed for paraffin blocks or whole mount analysis.

For the tumor studies heterozygous TTR-IGF-I (tg/wt) mice were crossed with heterozygous MMTV-ErbB2 (tg/wt) mice. The resulting offspring had one of 4 genotypes; heterozygous TTR-IGF-I (tg/wt), heterozygous MMTV-ErbB2 (wt/tg), bigenic TTR-IGF-I/MMTV-ErbB2 (tg/tg), or wild type controls (wt/wt). Female were weaned at 21 days and housed five per cage and monitored for mammary gland tumorigenesis. In all studies age-matched wild type littermates were used as controls.

Mice were palpated twice weekly to determine time to tumor formation, and once palpated, the rate of tumor growth was determined by measuring the tumor size with caliper measurements (millimeters/mm) and using the formula for an ellipsoid sphere: L × W^2^/2 = mm^3^. Mice were sacrificed when tumors reach 1000 mm^3^. Once tumors reached 1000 mm^3^, mice were injected with BrdU (100 mg/kg) for 2 hrs, sacrificed; and normal mammary glands and mammary glands with tumors were processed for paraffin blocks or frozen in liquid nitrogen.

### Whole gland morphological and histological analysis

For tumor and developmental studies mammary gland whole mounts were processed as previously described by Williams and Daniel [25] with the following modifications. The #4 inguinal mammary glands from the right side were removed and spread flatly on the inner surface of a 50 ml tube and fixed with with 10% Formalin in PBS. The next day, tissue was placed in a cassette and fat was removed using acetone for 48 hrs. Samples were dehydrated in 100% ethanol (EtOH) for l hr, 95% EtOH for 1 hr, and stained with Carmine Alum. Mammary glands were destained as follows: H_2_0 for 1 hr.; 70% EtOH for 1 hr.; 95% EtOH for 1 hr.; 100% EtOH 3 × for 1 hr.; and cleared in xylene 3 × for 1 hr. Finally tissues were permanently stored in glass vials filled with methylsalicylate until analyzed.

Percentage of fat pad filled was determined by measuring (mm) the length of ductal out growth of #4 inguinal normal mammary glands and dividing it by the length of the total length of the mammary gland fat pad at 4, 6 and 9 weeks of age.

Mammary gland tumors and #4 inguinal normal mammary glands were harvested, placed in cassettes and fixed in 4% paraformaldehyde in PBS overnight. The following day, paraformaldehyde was replaced by 70% EtOH and samples were embedded in paraffin. Serial sections (5 μm thick) cut from paraffin blocks were placed on Superfost Plus slides (Fisher Scientific, Fair Lawn, NJ), deparaffinized, gradually hydrated and all sections stained with Hematoxylin-Eosin (H&E) and then examined microscopically.

### Immunoblot analysis

Frozen mammary glands were first crushed under liquid nitrogen using a metal pestle and mortar. Crushed tissue was lysed in TNESV buffer and 50 μg of tissue protein lysate was immuonblotted as described previously [26]. We used the following antibodies at the listed concentrations: Anti-P85 1:1000 (Upstate Group, Inc., Lake Placid, NY, USA), p-AKT 1:1000 (Cell Signaling Technology, Beverly, MA, USA), p-ERK1/2 1:1000 (Cell Signaling Technology, Beverly, MA, USA), AKT 1:1000 (Cell Signaling Technology, Beverly, MA, USA), ERK1/2 1:4000 (Upstate Group, Inc., Lake Placid, NY, USA), and β-actin 1:4000 (BD Biosciences, San Jose, CA, USA).

### Hormone Assays

IGF-I levels were measured in serum using rat/mouse IGF-I ELISA assay purchased from Immunodiagnostic Systems (Boldon, Tyne & Wear, UK). The assay sensitivity was 82 ng/ml. IGF-I levels measured in mammary gland tissue were measured by first crushing the tissue under liquid nitrogen using a metal pestle and mortar. Crushed tissue was then lysed in TNESV buffer and 10 μl of supernatant was assayed by a newly developed mouse IGF-I High Sensitivity (HS) ELISA assay from Immunodiagnostic Systems. The assay sensitivity was 2.8 ng/ml.

### Statistical Analysis

In the tumor studies, weights between animals that expressed the TTR-IGF-I transgene (TTR-IGF-I and TTR-IGF-I/MMTV-ErbB2 bigenics) and those that did not (wild type and MMTV-ErbB2 trangenics) were analyzed using ANOVA. All other analysis was done by unpaired Student's *t *test assuming random sampling. Probability values < 0.05 were considered to be statistically significant. The IBM PC programs INSTAT and PRISM software (GraphPad, San Diego, CA, USA) were used to calculate and graph the results. Time to tumor formation was analyzed using Kaplan-Meier survival curves [27] and compared using the generalized Wilcoxon test [28]. Tumor growth rates were analyzed by computing the individual growth curve measurements from time of primary tumor appearance to attaining a size of 500 mm^3^. Then the growth rates for each mouse were compared using a Student's *t *test.

## Results and Discussion

### A moderate increase in levels of circulating IGF-I doesn't alter pubertal mammary gland development

**Figure 1:**
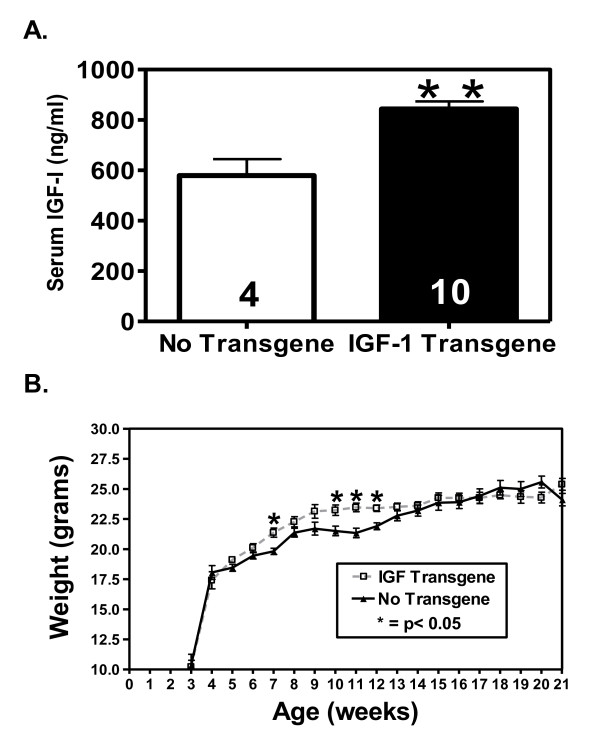
**Liver specific transgene overexpression of IGF-I results in increased levels of circulating IGF-I and increased body weight**. A.) Overexpression of liver IGF-I (black bar) resulted in a significant (p < 0.01) increase in female serum levels of IGF-I measured at 6 weeks of age compared to mice without the TTR-IGF-I transgene (white bar). B.) Females expressing the IGF-I transgene exhibited significantly (p < 0.05) larger body weights at 7, 10-12 weeks of age due to increased circulating IGF-I compared to wt/ErbB2 only controls. No transgene = wt + ErbB2 only females; IGF-I Transgene = TTR-IGF-I transgenic + TTR-IGF-I-ErbB2 bigenic females. N for each group is represented within their respective bars. N = 30 animals per group for weight. ** p < 0.01; * p < 0.05.

To examine the effect of elevated circulating IGF-I on pubertal mammary gland development and tumorigenesis, we crossed TTR-IGF-I heterozygous mice with MMTV-ErbB2 heterozygous mice. MMTV-ErbB2 mice are based on mammary specific overexpression of the ErbB2 receptor (a frequently amplified oncogene in human breast cancer), which results in mammary specific tumorigenesis [24]. The resulting offspring had one of 4 genotypes; increased systemic IGF-I only (Tg/wt), mammary specific ErbB2 overexpression only (wt/tg), both systemic IGF-I and mammary ErbB2 bigenic expression (tg/tg), or wild type controls (wt/wt). At 4 weeks of age there was a significant 9.5% increase in circulating IGF-I levels in TTR-IGF-I transgenic females (942.54 ± 21.57 ng/ml) compared to age matched controls (860.91 ± 20.79 ng/ml) (data not shown). At 6 weeks of age, TTR-IGF-I transgenic mice (TTR-IGF-I and TTR-IGF-I/ErbB2) showed a 35% increase in circulating IGF-I compared to mice without the TTR-IGF-I transgene (wt and ErbB2) (941.22 ±41.77 ng/ml vs. 743.69 ± 28.24 ng/ml - Figure 1A). As expected the ErbB2 transgene did not affect the TTR-IGF-I transgenic stimulation of increased circulating IGF-I in these animals. The increase in IGF-I caused a significant increase (p < 0.05) in overall body growth in animals expressing the TTR-IGF-I transgene during weeks 7, 10-12, however, body weight was equal afterwards (Figure 1B). Interestingly, a minor sustained increase in body growth in TTR-IGF-I trangenics was observed throughout the time of mammary gland development. We previously reported that homozygous TTR-IGF-I on a C57BL/6 genetic background had a 50% elevation of circulating IGF-I and resulted in a similar 10% increase in female bodyweight during weeks 8, 10-12 [21]. The lower level of circulating IGF-I in heterozygous TTR-IGF-I/FVB/N mice might be due to the use of heterozygous mice, or the FVB/N genetic background. The lack of a dramatic sustained growth difference in TTR-IGF-I/C57BL/6 or TTR-IGF-I/FVB/N is interesting and may reflect a threshold of IGF-I needed to observe a sizable difference. In a similar manner, previous studies shown that deletion of the liver IGF-I resulted in a dramatic reduction in circulating IGF-I but had no effect upon normal growth, and it wasn't until the acid labile subunit (ALS) was also deleted that levels went below a threshold needed to see an effect upon overall body growth [29,30].

**Figure 2:**
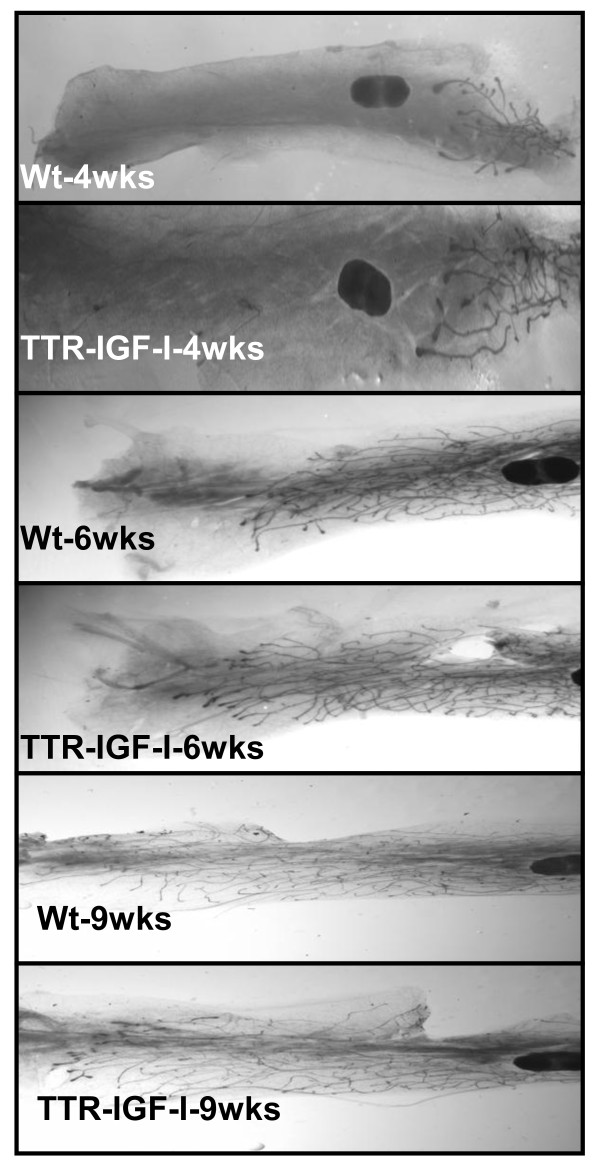
**Increased circulating levels of IGF-I had no effect on pubertal mammary gland growth**. Representative whole mounts of wild type (wt) and IGF-I transgenic (TTR-IGF-I) mammary glands at 4 wks (top 2 panels), 6 wks (middle 2 panels), and 9 wks of age (bottom 2 panels). N = 20 per group at 4 wks of age; N = 4 for WT and N = 7 for TG at 6 wks of age; N = 7 for WT and N = 8 for TG at 9 wks of age. WT = wild type and TG = TTR-IGF-I.

Whole mount analysis revealed that higher levels of systemic IGF-I had no effect upon the developing mammary gland compared to controls at 4, 6 and 9 weeks of age (Figure 2). More specifically, 26% of the mammary fat pad in the control (wt) and 28% in the TTR-IGF-I (tg) groups was filled with a developing ductal tree by 4 weeks of age. Similar ductal growth patterns between the two groups were also observed at 6 and 9 weeks of age, which is summarized in Table 1. The lack of effect of circulating IGF-I on mammary gland development is consistent with a previous studying showing that it is paracrine mammary gland IGF-I that is primarily responsible for ductal development [31]. Supporting this, ductal branching is significantly reduced in mammary glands from adult transgenic IGF-I^m/m ^mice which have a multi-tissue (including mammary gland) reduction in IGF-I production. However, in IGF-I LID transgenic mice (deletion of liver-specific IGF-I) mammary gland branching is unaffected by the 75% reduction in circulating IGF-I [32]. Thus, it is unclear how much circulating IGF-I is actually reaching the mammary gland. It is possible that IGF-I is bound up, by IGF-I binding proteins (BP), in the extracellular matrix of the mammary gland and is unable to bind to IGF-IR. In our initial characterization of the TTR-IGF-I mouse, liver overexpression of IGF-I was also associated with increased circulating levels of IGF-I BP3 [21], which may inhibit the ability of free circulating IGF-I to act on the mammary gland promoting growth and subsequent tumorigenesis. Interestingly, the hepatic IGF-I transgenic mouse (HIT) generated on the FVB/N background which has liver-specific production of circulating IGF-I (resulting in a 2.5-fold increase in circulation IGF-I without an increase in hepatic IGFI-BP3 or ALS [15] levels) has been shown to increase mammary gland proliferation and cause hyperplasia at 6 to 8 wks of age [16]. In the same study, Cannata *et al. *[16], showed that mice with transgenically increased circulating IGF-I, but no tissue IGF-I (KO-HIT), mammary gland proliferation was comparable to controls at 6 to 8 wks of age. Furthermore, the authors noted an increase in ductal branching at 4 and 16 weeks of age but not at 8 wks of age in HIT mice, whereas circulating IGF-I in KO-HIT mice can support mammary gland growth during this period without local IGF-I [16]. HIT mice have been shown to rescue developmental growth in IGF-I null mice but not correct reproductive deficiencies in these animals as a result of the absence of tissue IGF-I [15].These studies are compelling and support the notion that that circulating IGF-I has the ability to promote morphological changes in mammary gland development, particularly in the absence of local IGF-I. Importantly, no studies to date have shown that a systemic increase in circulating IGF-I can initiate and/or promote mammary tumorigenesis.

**Table 1:** Analysis of Mammary Gland Growth

Group	Wild Type (control)	Transgenic (TTR-IGF-I)
Average. % Fat Pad Filled *4 wks*	26%	28%

Average. % Fat Pad Filled *6 wks*	80%	87%

Average. % Fat Pad Filled *9 wks*	82%	82%

There was no difference in mammary gland development compared to age-matched wild types at 4, 6 and 9 weeks of age as determined by percent of the mammary fat pad filled with the ductal tree. N = 20 for WT and TG at 4 wks of age; N = 4 for WT and 7 for TG at 6 wks of age; N = 7 for WT and N = 8 for TG at 9 wks of age. WT = wild type and TG = TTR-IGF-I

### Increased circulating IGF-I doesn't affect mammary gland tumorigenesis

**Figure 3:**
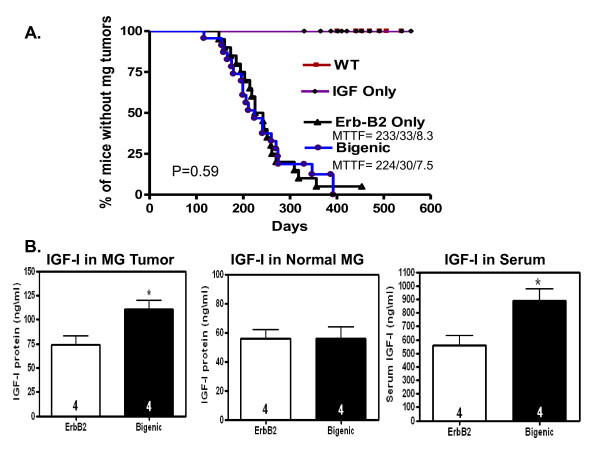
**Increased levels of circulating IGF-I does not initiate mammary tumorigenesis**. A.) Kaplan-Meier tumor curve illustrating the percent of animals without mammary gland tumors vs. the day tumors were first palpated. Increased IGF-I had no effect on ErbB2 initiated mammary tumorigenesis (p = 0.59). Additionally, the moderate increase in circulating IGF-I did not induced mammary tumors in females expressing only the IGF-I transgene (purple) compared to controls (maroon line). B.) Increased circulating levels on IGF-I resulted in significantly higher (p < 0.05) IGF-I protein levels in tumors from bigenic females compared to tumors in ErbB2 transgenics (right panel). Middle panel shows IGF-I protein levels measured in contralateral normal mammary glands from these same bigenic and ErbB2 transgenic females. Left panel shows a significant increase (p < 0.05) in serum IGF-I in these same bigenic females compared to ErbB2 transgenics. Bars indicate the mean (±SEM) serum or protein levels of IGF-I assayed by ELISA. N = indicated with bars; MG = Mammary Gland; ErbB2 = ErbB2 transgenic only; Bigenic = TTR-IGF-I-ErbB2; MTTF (mean time to tumor formation) = weeks/days/months; * = p < 0.05.

Bigenic IGF-I/ErbB2 virgin female mice showed palpable mammary tumors beginning at 24 weeks of age and had a mean time to tumor formation (MTTF) of 33 weeks (Figure 3A). ErbB2 only transgenic virgin females showed a similar tumor formation with the earliest mammary tumors palpated at 24 weeks of age and a MMTF of 30 weeks (Figure 3A). There was no difference in MTTF (p = 0.59). Importantly, increased circulating levels of IGF-I had no affect on mammary tumor incidence, as no mammary tumors were detected in the TTR-IGF-I only mice (Figure 3A). While species variation in IGF-I signaling may play a role in our findings compared to epidemiological data that currently exists [3] a wealth of evidence confirms the common functionality of IGF-I signaling between humans and mice in both normal growth and development, and numerous human malignancies not just breast cancer [33].

**Figure 4:**
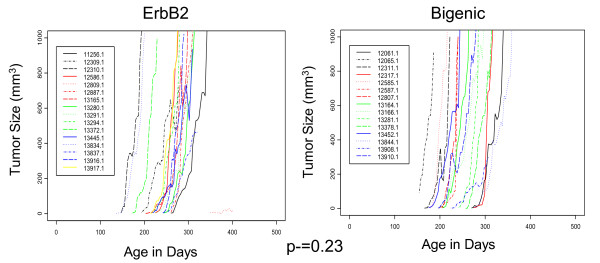
**Increased circulating IGF-I does not alter growth in ErbB2-induced mammary gland turmors**. Graphs plotting individual tumor growth curves for ErbB2 (left) and bigenic (right) females. Statistical analysis revealed that increased circulating levels of IGF-I (right) had no significant (p = 0.23) affect on mammary tumor growth compared to ErbB2 only induced mammary tumors. N = 16 for ErbB2 group and 15 for TTR-IGF-I-ErbB2 bigenic group.

Mammary tumors from bigenic mice had significantly (p < 0.05) higher bioavailable IGF-I protein levels (110.84 ± 9.02) compared to mammary tumors from ErbB2 only mice (73.97 ± 9.01) measured by ELISA (Figure 3B). This elevation mimicked the significantly increased (p < 0.05) circulating levels of IGF-I measured in these animals (Figure 3B) and were similar to the elevated levels in TTR-IGF-I animals at 6 wks of age (Figure 1A). However, there was no difference in IGF-I protein levels in the contralateral normal mammary glands (no detectable tumor) from the same animals (Figure 3B). The observed increase in IGF-I in the bigenic tumors might simply be a result of contamination from blood due to increased blood flow in the tumors. However, it is also plausible that the TTR-IGF-I transgene was activated in the tumors resulting in increased paracrine IGF-I protein levels which may have contributed to the measured increase of IGF-I in these tumors. Given this, we carefully examined the growth rate of the tumors to test if the increased IGF-I might alter tumor progression. Therefore the rate of tumor growth was assessed by comparing the tumor size (mm^3^) vs. the age in days of all individual tumors in both the ErbB2 (left panel) and bigenic groups (right panel; Figure 4). However, Figure 4 clearly illustrates that increased levels of IGF-I had no effect on the growth of ErbB2 tumors in these animals (p = 0.23).

A complete analysis of the groups revealed that 95% (19 of 20) of the bigenics and 90% (19 of 21) of the ErbB2 only transgenic mice developed mammary tumors. Furthermore, both groups of mice averaged approximately the same number of mammary tumors per animal (ErbB2 - 2.2 tumors/mouse and bigenic 2.3 tumors per mouse). This data is summarized in Table 2.

**Table 2:** Comparing Groups: End of study tumor summary

	ErbB2	Bigenic	IGF-I	Wild Type
Number of Tumors	19(20) = 95%	19(21) = 90%	0(25)	0(22)

Mean Time to Tumor Formation (MTTF)	224 days 30 wks/7.5 m	233 days 33 wks/8.3 m	NA	NA

Average Number of Tumors per Animal	2.2 (11) = 55%	2.3 (13) = 60%	NA	NA

Number of tumors = animals that got mammary tumors (total number of animals) %- percentage of animals with mammary tumors per group; Average number of tumors per animal = average number of mammary gland tumors per animal (number of animals with multiple tumors), %- percentage of animals with multiple tumors per group. NA = not applicable. ErbB2 = MMTV-ErbB2 transgenic; Bigenic = TTR-IGF-I-ErbB2 bigenic; IGF-I = TTR-IGF-I transgenic; Wild Type = control; wks = weeks; m = months.

**Figure 5:**
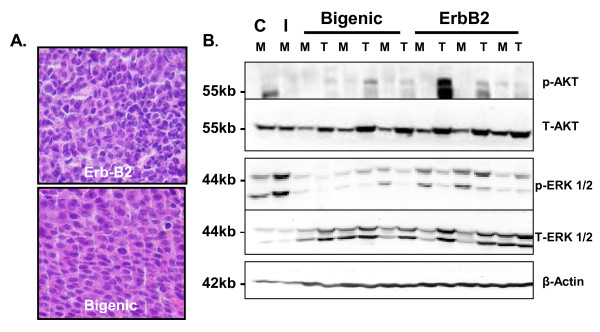
**Increased levels of circulating IGF-I have no effect on tumor type or mammary gland signaling**. A.) Representative H&E staining of mammary tumors promoted by MMTV-ErbB2 (top) or TTR-IGF-I-ErbB2 bigenic (bigenic-bottom). Both groups had similar tumor phenotypes which were predominantly adenocarcinomas. B.) Representative immunoblot of phosphorylated (p) and total (T) AKT and Erk ½ signaling in normal mammary glands (M) and tumors (T). *** = p < 0.001; Magnification: 40 ×. C = representative wild type females; I = representative TTR-IGF-I transgenic females

Whole mount (data not shown) and H&E analysis revealed no major morphological differences in ductal branching and tumor type (solid adenocarcinomas) between bigenic and ErbB2 mammary glands at the time tumors were harvested (Figure 5A). Furthermore, immunoblot analysis revealed that circulating levels of IGF-I seemingly had no effect on mammary gland signaling (Figure 5B). As expected, AKT phosphorylation was elevated in mammary gland tumors from both bigenic and ErbB2 females. There were no observable differences in ERK1/2 protein expression in mammary gland tumors from either group. More importantly, increased circulating IGF-I had no effect on normal mammary gland signaling compared to groups that did not express the TTR-IGF-I transgene (Figure 5B). Our findings are similar to a previous report showing that elevated levels of circulating IGF-I in HIT mice did not alter IGF-I regulated downstream signaling pathways [16].

## Conclusions

Epidemiological data suggests that increased circulating IGF-I is associated with a women's risk of developing breast cancer [3]. On the other hand, experimental evidence that increased circulating IGF-I is able to initiate and/or regulate tumor growth has not yet been established. Using the first transgenic animal model to simulate circulating levels of IGF-I that may be comparable to levels in women susceptible to breast cancer, our data suggest that modest elevation of circulating IGF-I do not have a role in mammary tumor initiation or promotion. More so, we showed that circulating IGF-I does not alter normal pubertal mammary gland development; thus supporting the established dogma that paracrine/autocrine IGF-I regulated by GH is the preferred regulatory pathway responsible for mammary gland development [34].

The inability of increased circulating IGF-I to initiate mammary tumorigenesis in our model suggest that the breast cancer risk associated with higher levels of circulating IGF-I in women may, in part, be due to IGF-I being associated or modulating another risk factor for breast cancer. Thus IGF-I may enhance a women's sensitivity to oncogenic promoters like ER (estrogen receptor) or ErbB2. IGF-I has been shown to increase transcriptional activation of ER in breast cancer cell lines increasing cellular sensitivity to the actions of estrogen [35,36]. However, estrogen has been shown to activate the IGF pathway in MCF7L xenographs independent of the level of circulating IGF-I [37]. Furthermore, we showed that increased circulating IGF-I levels had no effect on ErbB2 promoted mammary tumorigenesis. This would suggest the local interaction between the between the ErbB2 and IGF-I pathways previously shown [9,38] is independent of circulating IGF-I and more likely dependent upon alterations in autocrine/paracrine signaling pathways in the breast.

This work culminates to suggest that circulating IGF-I itself may not be directly altering breast cancer risk and thus may not be a suitable target for successful treatment. Further studies are required in additional models to determine if this result is common across other breast cancer subtypes.

## Abbreviations

IGF-I: Insulin-like growth factor-I; GH: Growth hormone; HIT: Hepatic IGF-I transgenic; KO-HIT: knockout mice expressing the hepatic IGF-I transgene; LID: Liver IGF-I Deficient; Lit/lit: *little *transgenic mouse; Tg: transgenic; Wt: wild type; mm: millimeters; EtOH: ethanol; H&E: Hematoxylin-Eosin.

## Competing interests

The authors declare that they have no competing interests.

## Authors' contributions

RKD did all the animal work, tumor palpations, developmental study, contributed to experimental design and drafted the manuscript. IK did all Immunoblot signaling analysis and contributed to manuscript preparation. YW participated in mammary gland immunohistochemistry (IHC) and contributed to manuscript preparation. LL generated the TTR-IGF-I transgenic mouse line. SGH did the statistical analysis and figure for tumor growth rates.

PHB provided ErbB2 animals for the study and aided in revisions of the manuscript. JX designed and provided TTR-IGF-I animals for the study, and aided in revisions of the manuscript. AVL provided project idea, contributed to experimental design and aided in writing of manuscript. All authors read and approved the final manuscript.

## Pre-publication history

The pre-publication history for this paper can be accessed here:

http://www.biomedcentral.com/1471-2407/11/377/prepub
